# Determining our climate policy future: expert opinions about negative emissions and solar radiation management pathways

**DOI:** 10.1007/s11027-022-10030-9

**Published:** 2022-10-03

**Authors:** Benjamin K. Sovacool, Chad M. Baum, Sean Low

**Affiliations:** 1 Aarhus University, Aarhus, Denmark; 2 Science Policy Research Unit (SPRU), University of Sussex, Jubilee Building, Room 367, Falmer, BN1 9SL East Sussex UK; 3 Boston University, Boston, USA

**Keywords:** Expert survey, Climate engineering, Carbon dioxide removal, Negative emissions technologies, Solar radiation management, Greenhouse gas removal

## Abstract

Negative emissions technologies and solar radiation management techniques could contribute towards climate stability, either by removing carbon dioxide from the atmosphere and storing it permanently or reflecting sunlight away from the atmosphere. Despite concerns about them, such options are increasingly being discussed as crucial complements to traditional climate change mitigation and adaptation. Expectations around negative emissions and solar radiation management and their associated risks and costs shape public and private discussions of how society deals with the climate crisis. In this study, we rely on a large expert survey (*N* = 74) to critically examine the future potential of both negative emission options (e.g., carbon dioxide removal) and solar radiation management techniques. We designed a survey process that asked a pool of prominent experts questions about (i) the necessity of adopting negative emissions or solar radiation management options, (ii) the desirability of such options when ranked against each other, (iii) estimations of future efficacy in terms of temperature reductions achieved or gigatons of carbon removed, (iv) expectations about future scaling, commercialization, and deployment targets, and (v) potential risks and barriers. Unlike other elicitation processes where experts are more positive or have high expectations about novel options, our results are more critical and cautionary. We find that some options (notably afforestation and reforestation, ecosystem restoration, and soil carbon sequestration) are envisioned frequently as necessary, desirable, feasible, and affordable, with minimal risks and barriers (compared to other options). This contrasts with other options envisaged as unnecessary risky or costly, notably ocean alkalization or fertilization, space-based reflectors, high-altitude sunshades, and albedo management via clouds. Moreover, only the options of afforestation and reforestation and soil carbon sequestration are expected to be widely deployed before 2035, which raise very real concerns about climate and energy policy in the near- to mid-term.

## Introduction

Carbon dioxide removal is coming to be viewed as important if not essential for reducing global temperate change or meeting the longer-term targets embedded in the Paris Accord (IPCC 2018). A strong majority of integrated assessment modeling scenarios discuss the widespread use of bioenergy with carbon capture and storage for meeting net-zero targets, finding that it could absorb more than 1000 GtCo2 between now and the end of the century, essentially doubling the carbon budget available to human society (Fuss et al. 2014). Its deployment is also seen as a cost-effective, climate-neutral opportunity in policy regimes such as the USA (Sanchez et al. 2015) or Sweden (Ministry of the Environment 2020). Other carbon dioxide removal techniques such as afforestation or soil management can enhance carbon uptake and be implemented more quickly than the time it takes to build some climate-mitigation actions (such as building large-scale nuclear power plants) (Houghton et al. 2015). Enhanced weathering could draw down atmospheric levels of carbon to the point where ocean acidification is effectively ameliorated by the end of the century (Taylor et al. 2016).

Other researchers have argued that humanity must seriously consider solar radiation management as a geoengineering technique to better address climate change (Keith 2013; National Academies of Sciences Engineering, and Medicine 2021). For example, stratospheric aerosol injection could serve as an emergency measure to slow the risk of global warming or create a stop-gap period of adjustment that gives countries time to adapt to the impacts of climate change (Barrett et al. 2014). Other options such as marine cloud brightening or cirrus cloud thinning could reduce the risk of pending “tipping points” in the climatic system, and diversify the portfolio of options we have to arrest suspected increases in temperature (Sovacool 2021).

Collectively, a surprising abundance of both carbon removal and solar radiation management techniques are available for consideration. On the negative emissions and carbon removal side, these include the 10 options in Table 1 (Sovacool 2021; Low et al. 2022a; Sovacool et al. 2022). On the solar radiation management side, these include the other ten options in Table 1 (Baum et al. 2022; Low et al. 2022b).

**Table 1:** Introducing 20 negative emissions and solar geoengineering options

Negative emissions and carbon removal	Solar radiation management and geoengineering
Afforestation and reforestation	Stratospheric aerosol injection
Soil carbon sequestration	Marine cloud brightening
Biochar	Cirrus cloud thinning
Bioenergy with carbon capture and storage (BECCS)	Space-based (extra-terrestrial) reflectors
Enhanced weathering	Albedo modification via human settlements
Ocean alkalinization or fertilization	Albedo management via grasslands and crops
Blue carbon and seagrass	Albedo management via deserts
Ecosystem restoration	Albedo management via clouds
Direct air capture and storage (DACCS)	Ice protection
Carbon capture utilization and storage (CCUS)	High altitude sunshades

Source: Authors

Despite the increasing importance of these 20 combined options in the recent literature, they remain highly contested. Large-scale deployment of bioenergy with carbon capture and storage would necessitate significant changes in land use including potential interference with agriculture and cropland (Obersteiner et al. 2018). Other strategies such as DACCS or enhanced weathering face barriers related to adequate underground storage of carbon dioxide as well as competition with biodiversity protection (Vuuren et al. 2018). Afforestation and reforestation efforts face significant issues concerning their permanence, negative impacts on local communities by losing access to common resources, and biodiversity impacts of monocultures (to name only a few) (Thomas et al. 2010; Galik et al. 2016; Dutschke et al. 2005). In the extreme, critics suggest that negative emissions and radiation management options could promote authoritarianism (Michaelowa 2021), or create a dangerous moral hazard that accelerates emissions (and consequent climate impacts) because policymakers believe foolhardily they no longer need to mitigate emissions deeply or quickly (Anderson and Peters 2016; Bellamy 2018; Vuuren et al. 2017). Some academics have even called for a treaty of “non-use” that would prohibit the global deployment of solar geoengineering (Biermann et al. 2022). Decarbonization options as a whole also face a broad array of sociotechnical barriers spanning misaligned behavioral attitudes and practices, poorly developed business models, lack of policy guidance, and resistance from incumbents (Geels et al. 2017).

What is a policymaker to do? Expectations around negative emissions and solar radiation management and their associated risks and costs shape formal and informal responses to the climate crisis. In this study, we rely on a large expert survey exercise to critically examine the perceived feasibility of both negative emission options (e.g., carbon dioxide removal) and solar geoengineering options (e.g., solar radiation management). We designed an expert survey that asked a pool of 74 prominent experts questions about (i) the necessity of adopting negative emissions or solar radiation management options, (ii) the desirability of such options when ranked against each other, (iii) estimations of future efficacy in terms of temperature reductions achieved or gigatons of carbon removed, (iv) expectations about future scaling, commercialization, and deployment targets, and (v) potential risks and barriers. These five dimensions are relevant given they cover some of the most pressing challenges and debates facing carbon removal and solar radiation management, namely concerns about whether, how, at what cost, and when deployment should occur, as well as other concerns that may arise if and when deployment happens. To further justify these different dimensions, we engage with the extant literature on them in each of the five subsections to better demonstrate findings but also compare and contrast our own from the existing body of evidence.

Our primary contribution is both to report the results of the first expert survey we know of examining the full suite of negative emissions and solar radiation management options (unlike elicitations looking at one specific technology or pathway in isolation, e.g. (Dai et al. 2021; Vaughan and Gough 2016)), and to create an established baseline of expert opinion which can serve as useful benchmark by which to evaluate deployment and diffusion, including assumptions embedded into Integrated Assessment Models (Anderson and Jewell 2019; Braunreiter et al. 2021; Pielke and Ritchie 2021). We provide original data and analysis about opinions on the complementarity of these options, but also potential risks concerning individual and collective deployment.

## Research design

Our research design centered on a survey of expert opinion, adapted for personal safety during the COVID-19 pandemic (done via an online medium, Zoom). This approach has connections to expert elicitation, although our study does not meet the full requirements for expert elicitation, as we will explain below. But to provide some context, expert elicitation involves a decision-science approach calling on “experts”—those with well-established knowledge and judgments on a given topic—to identify relevant factors and support decisions being made by private actors or public policymakers. Expert elicitation can make a valuable contribution to informed decision-making (Morgan 2014). It has advantages over other forms of qualitative data collection or stated preference techniques given it tends to produce high-quality, transparent, and traceable knowledge on parameters for which there is no established expert consensus (Usher and Strachan 2013). Expert elicitation can be particularly effective when utilized to assess new or emerging technologies with high rates of uncertainty (and possible forecasting bias) and a lack of agreement about cost and performance (Abdulla et al. 2013; Anadon et al. 2016), a situation that we believe certainly applies to both negative emissions technologies and solar geoengineering (Sovacool 2021; Grant et al. 2021). Elicitation can finally offer valuable input into other techniques (that can build on it) such as scenarios or forecasts (Wiser et al. 2021).

We call our study an expert survey because it does not meet the full requirements for a formal expert elicitation. Some of the most intensive expert elicitations occur over hours to days of time together as a group where experts deliberate through multiple rounds of “elicitation” to identify consensus, or areas of dissensus. More formal expert elicitations must specify whether they are eliciting preferences or parameters, which are distinct elements. In our survey, we only did one “round” of elicitation (the survey), in isolation (each expert completed the instrument by themselves). Moreover, our survey involves both preferences and parameters. For instance, we ask our experts to identify their preferred temperature targets, one that limits climate change to what they would consider a non-dangerous level. This is not a factual question, but a matter of preference. What is considered dangerous will vary from expert to expert and depends on their preferences. Leaving our questions open like this also makes deciphering parameters difficult, and it suggests we do not meet the full criteria for a proper elicitation.

Our expert survey process involved selecting a pool of prominent experts, and then arranging over Zoom for them to complete our survey instrument (shown in Appendix 1). This questionnaire focused on different dimensions of negative emissions and solar geoengineering technologies, with topics including the necessity and desirability of interventions, their efficiency and feasibility, expected timings about scaling and commercialization, and concerns about risks and barriers. As is apparent in Appendix 1, our instrument relied on a range of forced-choice questions (requiring yes/no answers), ranking questions (requiring respondents to rank options against each other), Likert-scale questions (requiring respondents to assign a weighted answer to a question), and some open-ended questions (asking for respondents to input expected values related to things like date of commercialization or cost). Our use of “the best–worst scaling methods” as well as ranking and rating approaches offers an optimal technique to describe the relative desirability of the various options, given that they actively ask respondents to choose best, second-best, worst, second-worst (and so on) options (Erdem et al. 2012; Caputo and Lusk 2020; Jaeger et al. 2008).

Our expert survey focused on the ten different negative emissions technologies (grounded in the literature) as well as ten different solar engineering options (grounded in the literature) mentioned in the Introduction, for a total of twenty options.^1^ To be clear, focusing simultaneously on both carbon dioxide removal and solar radiation management is controversial. Nevertheless, there is a case to be made for looking at them comprehensively, as some studies have done (Delina 2021; Honegger et al. 2021a, b). The nature of our funding and premise of our entire GENIE project was to offer comparative analysis, we have been explicitly funded to look at the full portfolio of climate protection and geoengineering pathways, without bias or predetermined conclusions about them. Our broad approach across carbon dioxide removal (CDR) and solar radiation management (SRM) technologies is matched to our data collection techniques, we asked respondents about all options. That said, respondents could narrow their answers to only one or a few options, although they were not prompted to do so. That is, we did not force respondents to be either narrow or broad—we left the focus to them to which questions they felt competent enough to answer. Many raised issues of splitting vs. lumping, and many also pointed out that the same risks or actors or venues emerge across different CDR or SRM (or mitigation or adaptation) approaches. Moreover, our project adheres to the “matching principle” in environmental law (Butler and Macey 1996), which suggests the scale of a solution ought to match the scope of the problem, there is therefore an urgent social need to examine trade-offs within multiple options and across pathways. Lastly, our approach investigating CDR and SRM has strong relevance to policy recommendations, as it mirrors the policymaking dilemma of choosing options with limited resources and uncertainty. In the words of one of our respondents, “nothing is more important for climate policy” than understanding how CDR and SRM options might work together, or not.

Our recruitment and sampling of experts focused on a mix of advocates and critics, although we invited only those who have published peer-reviewed research papers on the topic, or published patents and intellectual property, within the past ten years (from 2011–2020). The lead author approached 125 experts via email to participate in our study, with 74 agreeing to take part (a response rate of 59.2%). We then distributed our instrument to these experts closely associated with negative emissions and/or solar geoengineering research or commercialization over the course of May to August 2021. Table 2 shows an overview of the demographics of our sample, and Appendix 2 lists all 74 experts who participated. Note that in some cases, experts did not answer every question (although each question still had a majority of experts answer it); for this reason, we describe specific respondent numbers in the captions of figures and data tables supplementing our analysis. This also hedges against an expert’s potential ability to *not* be comfortable answering questions or parts of our exercise by which they did not believe they had sufficient knowledge or experience; experts were encouraged only to answer questions by which they had sufficient expertise to address. For this reason, the paper actively describes the number of experts that answered each question (and it can be taken as an additional measure of self-reported knowledge literacy among respondents, in that they are only providing answers for questions which they believed they were an expert in).

**Table 2:** Summary of the demographics of experts who took part in our survey

Summary information	No
No. of experts	74
No. of organizations represented	63
No. of countries represented	15
Cumulative years spent in innovation or research of negative emissions and/or solar geoengineering	810
Average years spent in innovation or research of negative emissions and/or solar geoengineering	6.8
No. of experts whose current position falls into the following areas:	
Civil society and nongovernmental organizations	8
Government and intergovernmental organizations	4
Private sector and industrial associations	6
Universities and research institutes	56

Authors. Note: Appendix 1 shows precisely which experts had multiple roles, straddling constituencies

One notable limitation to our sample of experts, given that they had to have published in the peer-reviewed literature on negative emissions or solar geoengineering technologies, is that they do reflect existing biases in the research community. There is for instance a strong overrepresentation of experts from the United States (about 41%) and the Global North, and only a small number of experts from Africa and Asia. This does mean that our sample does not adequately represent the view of those in the Global South, an established problem within this body of research (Biermann and Möller 2019). Furthermore, many studies using qualitative data such as ours are not fully replicable, given that even repeating our research design precisely (but at a later time period) would face complications over the availability of experts (some might decline the invitation), the timeliness of answers (some might change their answers), and the adaptability of answers (some may have changed their views or thoughts since the time of the interview).

Moreover, we took an ethnographic approach that did not correct or problematize responses, so we present the unadjusted views of participants, even if they may have had misperceptions on specific points. This means our expert dataset is grounded on propositional knowledge under a situation of great uncertainty, and that our respondents are presenting their “justified belief” rather than any sort of objective fact (Sovacool et al. 2022). Indeed, one implication from our analysis is that no such objective fact or consensus exists concerning CDR and SRM options within our pool of experts. In simpler terms, respondent answers could be closer to “guesses” than “estimates.”

Finally, given the diversity of our expert sample, there is great variation in responses, signified further by large standard deviations when one quantitatively assesses our data (explored more in Appendix 3). That said, we are unable to correlate specific responses with individuals given that experts were participating in the study on the grounds that their identity would be kept *completely* confidential, that is without any identifiers, including gender, location, or affiliation.

## Results

Our results from the expert survey are associated with five broad themes.

### Necessity of interventions

One area of debate within the literature concerns the necessity of relying on negative emissions technologies and/or solar geoengineering as climate-policy options. One line of thinking strongly opposes their consideration at all, on the grounds that they are too risky (perpetuating a “risk–risk” tradeoff, that is, that some risks are addressed only by creating other risks (National Academies of Sciences Engineering, and Medicine 2021)), that they introduce a moral hazard (and are prone to “mitigation deterrence” that will interfere with carbon abatement options (The Royal Society 2009; Strefler et al. 2018; Vuuren et al. 2017; Preston 2011; Anderson and Peters 2016; McLaren 2020), or that they are extremely costly, energy intensive and/or not yet ready for deployment (Buck 2016; National Academies of Sciences Engineering, and Medicine 2019; Creutzig et al. 2019). An opposing line of thinking counters that prudence requires that society consider all potential options and hedge risk by seriously considering geoengineering approaches (Stephens and Keith 2008); that large-scale negative emissions technologies are absolutely essential for reaching 1.5 °C or 2° climate targets (Rueda et al. 2021; Gasser et al. 2015); and that delays in climate mitigation and underinvestment in adaptation demand that we pursue these options, (EASAC 2018; Jinnah and Nicholson 2019a; Jinnah and Nicholson 2019) as they “must be considered” (Nicholson et al. 2018).

**Fig. 1:**
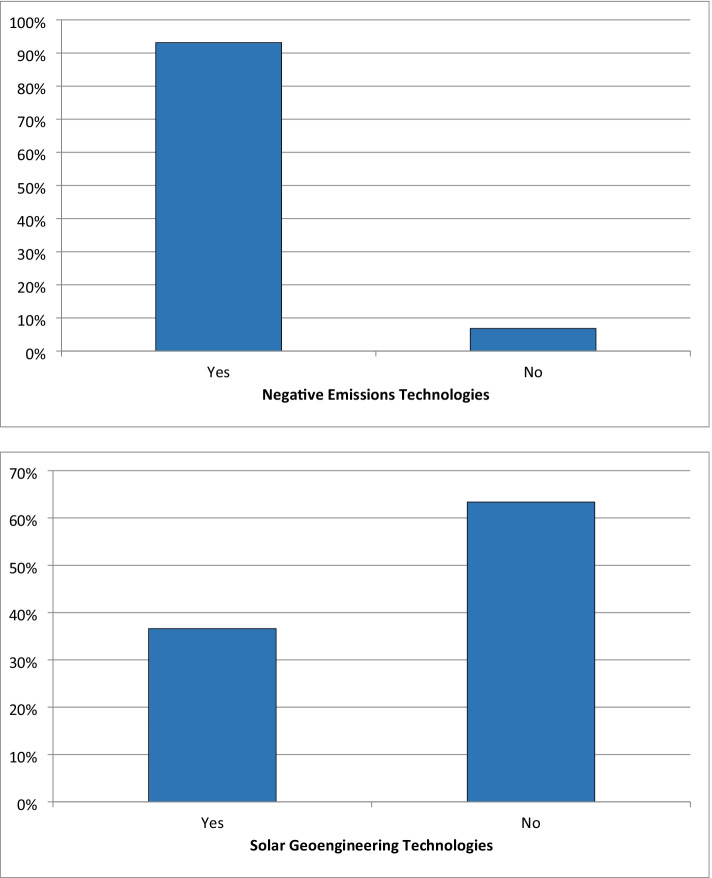
Expert opinions on the necessity of negative emissions (top panel) and solar geoengineering technologies (bottom panel). Source: Authors. The top panel (*N* = 73 respondents) depicts the answer to the question “Do you think that we will need greenhouse gas removal (GGR) and/or carbon dioxide removal (CDR) technologies in order to limit climate change to a non-dangerous level?” The bottom panel (*N* = 71 respondents) depicts the answer to the question “Do you think that we will need solar radiation management (SRM) technologies in order to limit climate change to a non-dangerous level?” We left it to each expert to self-determine their own standard of “need” when answering both questions. The full data tables behind this figure are presented in 4.3.

Our own results offer more nuance and depth to this discussion, showing (in Fig. 1) that our pool of experts strongly views negative emissions technologies as necessary to reach climate targets (top panel, more than 90%). Most of those supporting negative emissions come from universities and research institutes, governments, and the private sector; almost all of those that oppose (indicating no need) were from civil society institutions. However, the bottom panel shows that perceptions are inverted for solar geoengineering, with almost two-thirds of experts arguing that those options are not necessary. Remarkably, the strongest opposition comes from universities and research institutes along with governments and civil society.

### Desirability and comparative optimality of options

Negative emissions and solar geoengineering options do not exist in a vacuum, nor would they likely be deployed in isolation. Instead, many different technologies could be deployed simultaneously across multiple dimensions (e.g., enhanced weathering as well as direct air capture and BECCS, or sun shields with aerosol injection along with cloud brightening) as a form of “cocktail geoengineering,” (Long et al. 2017) or “portfolios” of negative emissions technologies (McLaren 2012; Reiner 2016). Despite the strong likelihood of such a diversified deployment pattern, the understanding within the literature of how to model, anticipate, and capture such complex portfolios and cocktails is currently limited, given that many models make overly simplistic assumptions about deployment (McLaren 2018; Butnar et al. 2020; Fuss et al. 2018; Low and Honegger 2020).

**Fig. 2:**
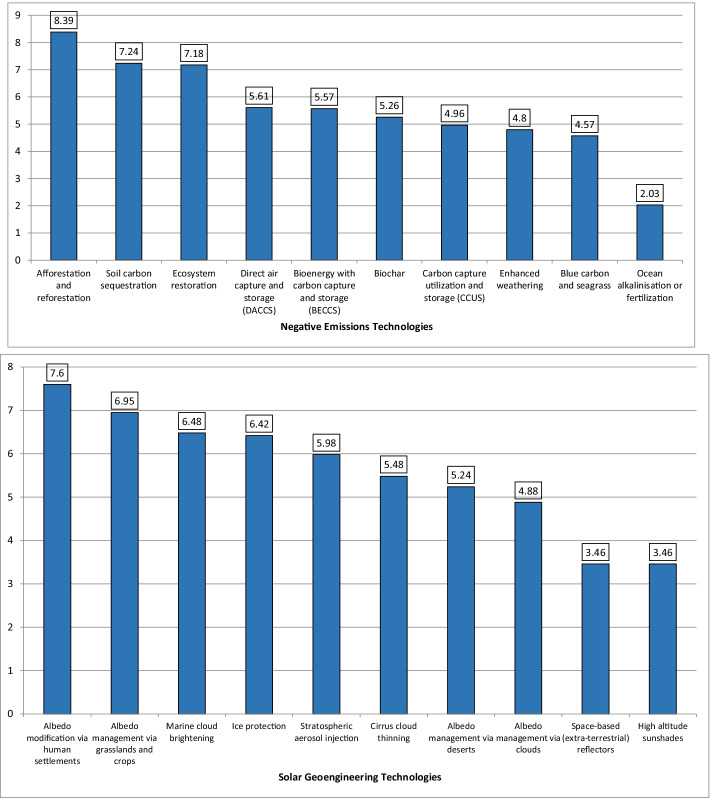
Expert perceptions on the preferred ranking of different negative -emissions (top panel) and solar -geoengineering options (bottom panel). Source: Authors. Note: the higher the number, the more positive the ranking (with one being the lowest and ten the highest). The top panel (*N* = 71 respondents) shows answers to the question “The literature on energy and climate policy often discusses the following GGR and CDR options. Please rank them against each other in order of your preference.” The bottom panel (*N* = 64 respondents) shows answers to the question “The literature often discusses the following SRM options. Please rank them against each other in order of your preference.” We left it to each expert to rank these options without any prompts or information treatments, meaning answers may reflect interest in or familiarity with the topic as much as any preference about research or deployment. The full data tables behind this figure are presented in Appendix 3

Our expert survey exercise was intended to tackle this gap, asking experts to rank in order of preference different options against each other, that is, comparatively. Looking at Fig. 2, the top panel shows negative emissions options grouped by their mean ranking across the expert survey, with Appendix 1 showing the precise questions asked. Afforestation and reforestation (mean rank of 8.39), ecosystem restoration (7.18), and soil carbon sequestration (7.24) are the most preferred by our experts, whereas ocean alkalinization or fertilization (2.03)—and, less so, blue carbon and seagrass (4.57), and enhanced weathering (4.8)—is by far the least preferred. Afforestation and reforestation being the most preferred for experts echoes findings for the general public, (Campbell-Arvai et al. 2017; Braun et al. 2018; Wolske et al. 2019; Jobin and Siegrist 2020; Shrum et al. 2020; Sweet et al. 2021), as does the generally positive view of ecosystem restoration. Though there is some disparity in the literature on soil carbon sequestration, the distinction by experts between this approach and other more technical options such as DACCS and BECCS is also becoming more evident for the public (Sweet et al. 2021; Wenger et al. 2021). Similarly, the lack of support for ocean fertilization, and indeed many ocean-based options—we note here that the second-lowest rating belonged to blue carbon and seagrass (4.57)—is another point of intersection between experts and the public (Jobin and Siegrist 2020; Shrum et al. 2020; Cox et al. 2020; Cox et al. 2021). The same also holds true for the broadly ambiguous and tentative perceptions for enhanced weathering at present (Wright et al. 2014; Pidgeon and Spence 2017; Spence et al. 2021). Appendix 3 shows more detailed statistical analyses of the data, and it moreover reveals how the highest standard deviation belongs to carbon capture storage and utilization, reflecting perhaps greater uncertainty and divergence of opinion, whereas the lowest standard deviation corresponds to afforestation and reforestation as well as ocean alkalinization or fertilization. The intermediate values for bioenergy with carbon capture and storage, direct air capture, and biochar furthermore offer a challenge to studies arguing that these options could or should become dominant negative emissions pathways by 2050 or 2100, e.g. (Fridahl and Lehtveer 2018; Buck 2019; Hanna et al. 2021; McQueen et al. 2021a, b; International Biochar Association 2021; Draper 2021).

The bottom panel of Fig. 2 reveals the stated rankings for solar geoengineering options (with the precise questions asked presented in Appendix 1). Albedo modification via human settlements (7.6) is the most preferred, followed by albedo management via grasslands and crops (6.95) and marine cloud brightening (6.48). Conversely, space-based reflectors (3.46), high-altitude sunshades (3.46), and albedo management via clouds (4.88) are the least preferred. Focusing on the relative preferences of the SRM options, we note a similar tendency between experts and the lay public for space-based approaches to rank lower (Jobin and Siegrist 2020) and, to some extent, for marine cloud brightening being preferable to stratospheric aerosol injection (Wright et al. 2014; Amelung and Funke 2015; Carlisle et al. 2020). Unlike the negative-emissions options, Appendix 3 reveals that standard deviations for these options are much, much higher, i.e., with the opinions of experts being much more divided. The standard deviation for stratospheric aerosol injection stands out as the highest of any of the options, whereas albedo management via clouds, conversely, is the lowest. Given that SRM options were ranked against one another, and not against CDR options, it is not possible to identify a pattern where SRM options such as stratospheric aerosol injection tend to be slightly less preferable, which is typical for public perceptions (Braun et al. 2018; Jobin and Siegrist 2020; Wright et al. 2014; Carlisle et al. 2020; Pidgeon et al. 2012; Bellamy et al. 2016; Merk et al. 2019; Klaus et al. 2020). At the same time, the greater heterogeneity of expert opinion, especially with regard to stratospheric aerosol injection, mirrors the tendency for public evaluations to vary depending on the amount of information provided, mode of discussion, and over time (Braun et al. 2018; Carlisle et al. 2020; Merk et al. 2019). This indicates that, for both experts and the lay public, the way that these options are viewed is still in flux, despite the greater knowledge possessed by experts. Indeed, one follow-up study (Carlisle et al. 2020), conducted 6 years after the original one (Wright et al. 2014), interestingly found a reverse in preference, with stratospheric aerosol injection having more negative associations than space-based approaches. Among other things, this suggests that greater knowledge and familiarity with options such as stratospheric aerosol injection could ultimately have an adverse effect on desirability.

### Estimations of efficacy and economic feasibility

We asked our experts to also quantify as best as they can the potential efficacy and feasibility of options in terms of achieved emissions reductions or successful temperature change, another area of great contestation within the literature (Fuss et al. 2018; National Research Council 2015; National Academies of Sciences Engineering, and Medicine 2021).

**Fig. 3:**
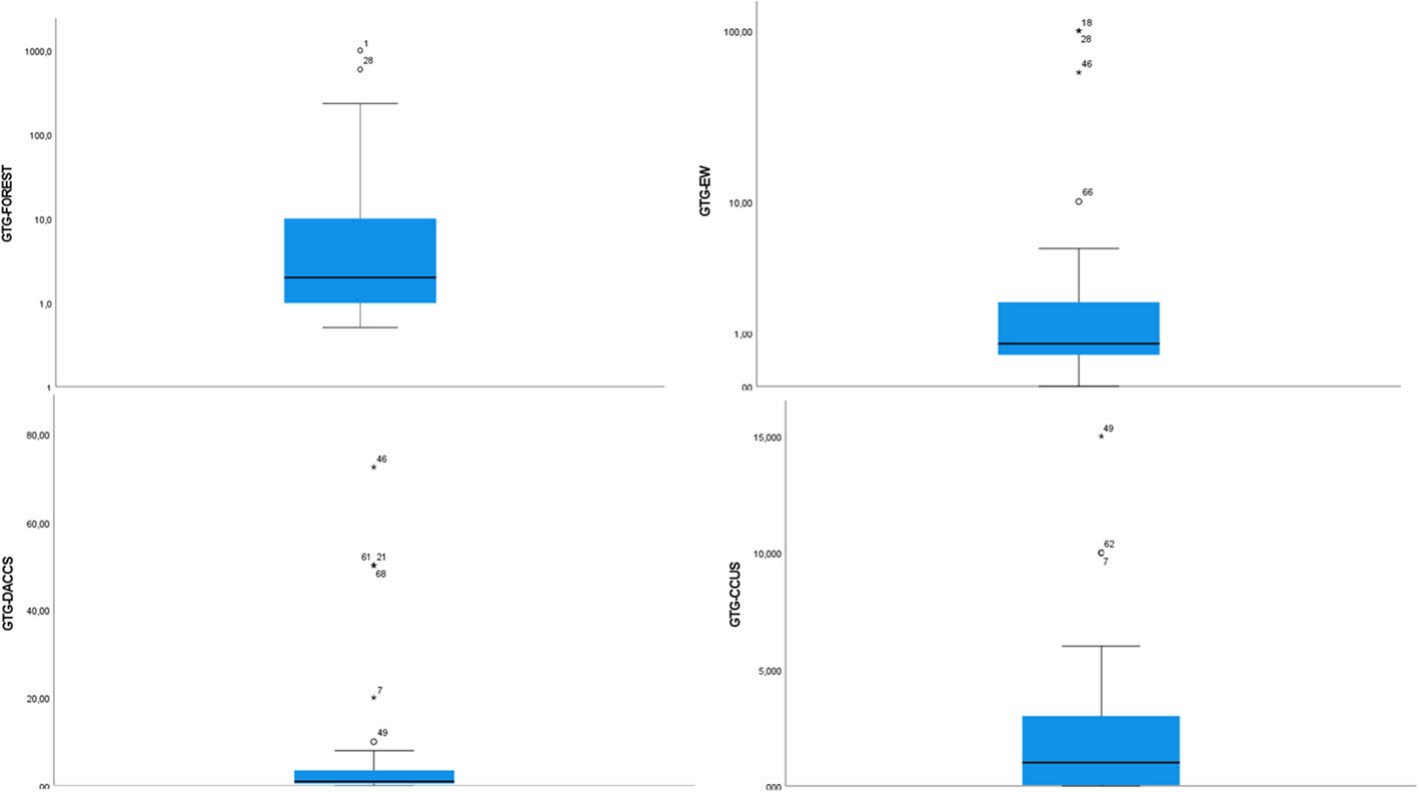
Expert perceptions on the greenhouse-gas-reduction potential of negative-emissions technologies. Source: Authors (*N* = 47 respondents). The figure shows the results (on a logarithmic scale, to include all outliers) for the question “Another way of prioritizing options is to quantify them. By the year 2050, how many gigatons of carbon dioxide equivalent do you expect each of the following options to reduce, displace, or avoid?” Illustrative results are shown for afforestation and reforestation, enhanced weathering, direct air capture, and carbon capture utilization and storage. The bars within the boxes refer to the median. As with our other questions, this one required our experts to judge for themselves expectations about future quantification. The answer depends entirely on how much the expert thinks this will be deployed, which may depend heavily on policies and goals, or other assumptions. The full data tables behind this figure are presented in Appendix 3

Figure 3 provides illustrative results in terms of the expected net gigatons of carbon dioxide selected negative emissions technologies could reduce, displace, or avoid by 2050. We prepared this question to supplement estimates based on integrated assessment modeling—with the key caveat that this literature admits many limitations. Fuhrman et al. (Fuhrman et al. 2019), summarizing the literature, note that scenarios “widely assume we are capable of scaling up NETs over the coming 30 years to achieve negative emissions of the same order of magnitude as current global emissions (tens of gigatons of CO2/year) predominantly relying on highly land intensive NETs.” Yet, IAM-calculated IPCC pathways have yet to comprehensively include engineered approaches, such as direct air capture and enhanced weathering, as well as a range of coastal or ocean-based approaches (O’Neill et al. 2020; Fuhrman et al. 2021). Emissions from supply chains and life cycles, from different carbon removal approaches, and across different geographies and timelines, also vary considerably, and have yet to be codified in any definitive way (Clery et al. 2021; Carton et al. 2021). Finally, there is the prospect of mitigation deterrence, which may considerably counterbalance the carbon removed, but in ways that have yet to be incorporated into IAMs (McLaren 2020).

As Appendix 3 reveals, the statistical data behind the results reveal that the three options with the most (mean) potential are afforestation and reforestation (mean of 61.250 GtCo2, median of 2 GtCo2), followed by ocean alkalinization or fertilization (58.9 Gt, 0 Gt) and bioenergy with carbon capture and storage (48.7 Gt, 2 Gt). The options with the least (mean) abatement potential were enhanced weathering (mean of 13.37 GtCo2, median of 0.75 Gt), carbon capture and storage (24.55 Gt, 1 Gt), and blue carbon and seagrass (26.34 Gt, 0.35 Gt). Given the expansive range of expert estimates, we report both mean and median values. Whereas the former gives a sense of the diversity of the estimations, the latter can be understood as a more consensus-based, less optimistic estimate of the options’ potential. Of note, if one combines the medians for all ten of the options, this amounts to 10.35 GtCo2—a pittance. The highest median value of any option is only 2 GtCo2, for afforestation, soil carbon sequestration, and bioenergy with carbon capture and storage—options with more near-term viability and deployment potential. In contrast, though ocean alkalinization or fertilization ranked as the second-highest option in terms of mean abatement potential, the median estimate is that it would not contribute at all to emissions reduction.

**Fig. 4:**
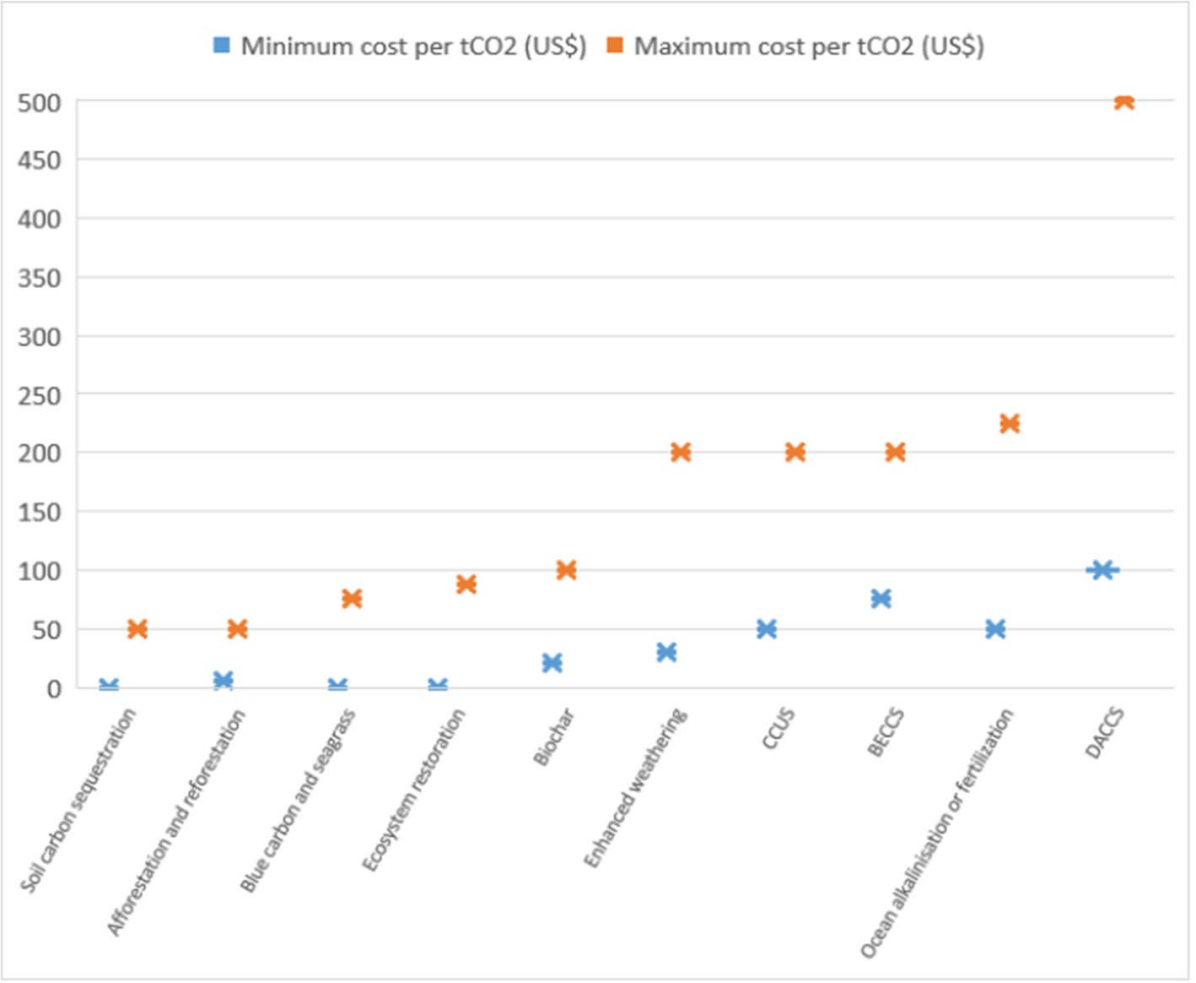
Expert perceptions on the efficacy and cost of negative-emissions technologies. Source: Authors (*N* = 45 respondents). The figure shows results for the question “Similarly, another way of considering options is according to their costs of carbon removal. The uncertainties are obviously very large, so providing a range of estimations is fine. How would you estimate the range of costs in US$ per metric ton of carbon dioxide avoided by 2050?” Experts were able to give whatever range they preferred, without consideration of percentiles or triangulation with the existing modeling literature. The full data tables behind this figure are presented in Appendix 3

Figure 4 depicts expected costs for carbon removal (in US$ per metric ton by 2050). This, again, is a very controversial point in the existing policy and academic literature, with cost estimate varying widely based on assumptions about future learning, economies of scale, supportive policy environments, and technical performance, among other factors (Fuss et al. 2018; Heutel et al. 2015; National Research Council 2015; National Academies of Sciences Engineering, and Medicine 2019; Parliamentary Office of Science and Technology 2017). Here we opted to use the medians instead of the means owing to the existence of a couple outliers (and significant impacts on standard deviations) tending to skew results (see Appendix 3), notably, leading to maximum cost estimates which would exclude any consideration of some of the options. Interestingly, results cluster according to three core groups. One collection of more natural- and land-based solutions all have the lowest expected costs of abatement, notably: soil carbon sequestration (range from $0 to $50 per ton/C02), ecosystem restoration ($0 to $87.50), afforestation and reforestation ($0 to $50), blue carbon and seagrass ($0 to $75), and biochar ($20 to $100). All of these have median maximum costs lower than $100 and median minimum costs lower than $20, with most at or near zero. This contrasts with a second clustering of options that see expected minimum costs of at least $30 (in the case of enhanced weathering) and expected maximum costs less than $225—this class includes enhanced weathering (with a range from $30 to $200), carbon capture and storage ($50 to $200), ocean alkalinization or fertilization (€50 to $225), and bioenergy with carbon capture and storage ($75 to $200)). And lastly, in a class of its own, is direct air capture with median expected costs ranging from $100 to $500.

**Fig. 5:**
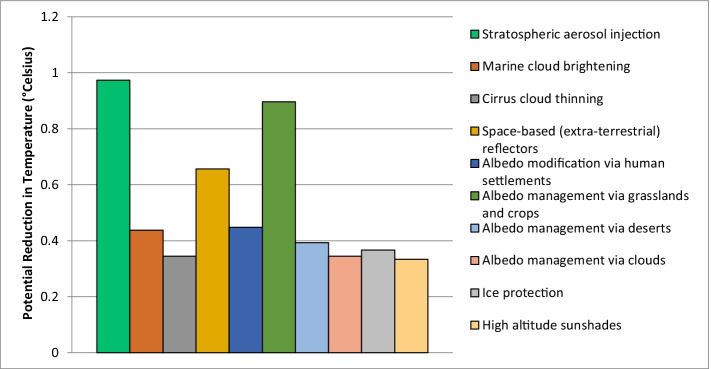
Expert perceptions on the temperature-reduction potential of solar-geoengineering technologies. Source: Authors (*N* = 40 respondents across the entire sample, although specific estimations for specific options vary and are reported in Appendix 3). Experts were able to give whatever range they preferred, without consideration of percentiles or triangulation with the existing modeling literature. The figure shows the results for the question “In terms of feasibility, by the year 2050, how much global warming or climate change (in degrees Celsius) do you expect each of these options to achieve reducing or addressing?”

Because the efficacy of solar geoengineering options tends to be assessed in degrees of temperature change rather than tons of carbon abatement, Fig. 5 plots our expert survey data according to how much global warming or climate change (in degrees Celsius) solar radiation management techniques are expected to achieve. As our data indicates, all options have a median value of 0°. However, when looking at the mean results, which provides a sense of development potential (and difference of opinion among experts) stratospheric aerosol injection is seen as the most promising, with the ability to avoid almost 1 degree of temperature change Celsius by 2050. This is followed in order of efficacy by albedo management via crops (a mean of 0.89° change) and space-based reflectors (0.656° change). The options deemed the least effective were high altitude sunshades (0.333° change), cirrus cloud thinning (0.344° change), and albedo management via clouds (0.344° change). Appendix 3 reveals the underlying statistical data behind these means, and it also shows the high frequency with which many respondents actually assigned a value of “0”—in many instances this occurred in a strong supermajority of responses.

### Expectations of scaling and deployment

Another core theme of our expert survey related to scaling and future deployment. We asked our experts to explicitly consider the specific year (between now and the end of the century) they expect options to achieve “widespread deployment,” which we inferred to mean at least a market share of 20% (a threshold drawn from some recent debates on energy-transition dynamics, historical diffusion of energy systems, and debates on energy system transformation (Grubler et al. 2016; Sovacool 2016)). The literature confirms that this issue of timing is incredibly important to deployment efficacy and achievability (Richard et al. 2021; MacMartin et al. 2021).

**Fig. 6:**
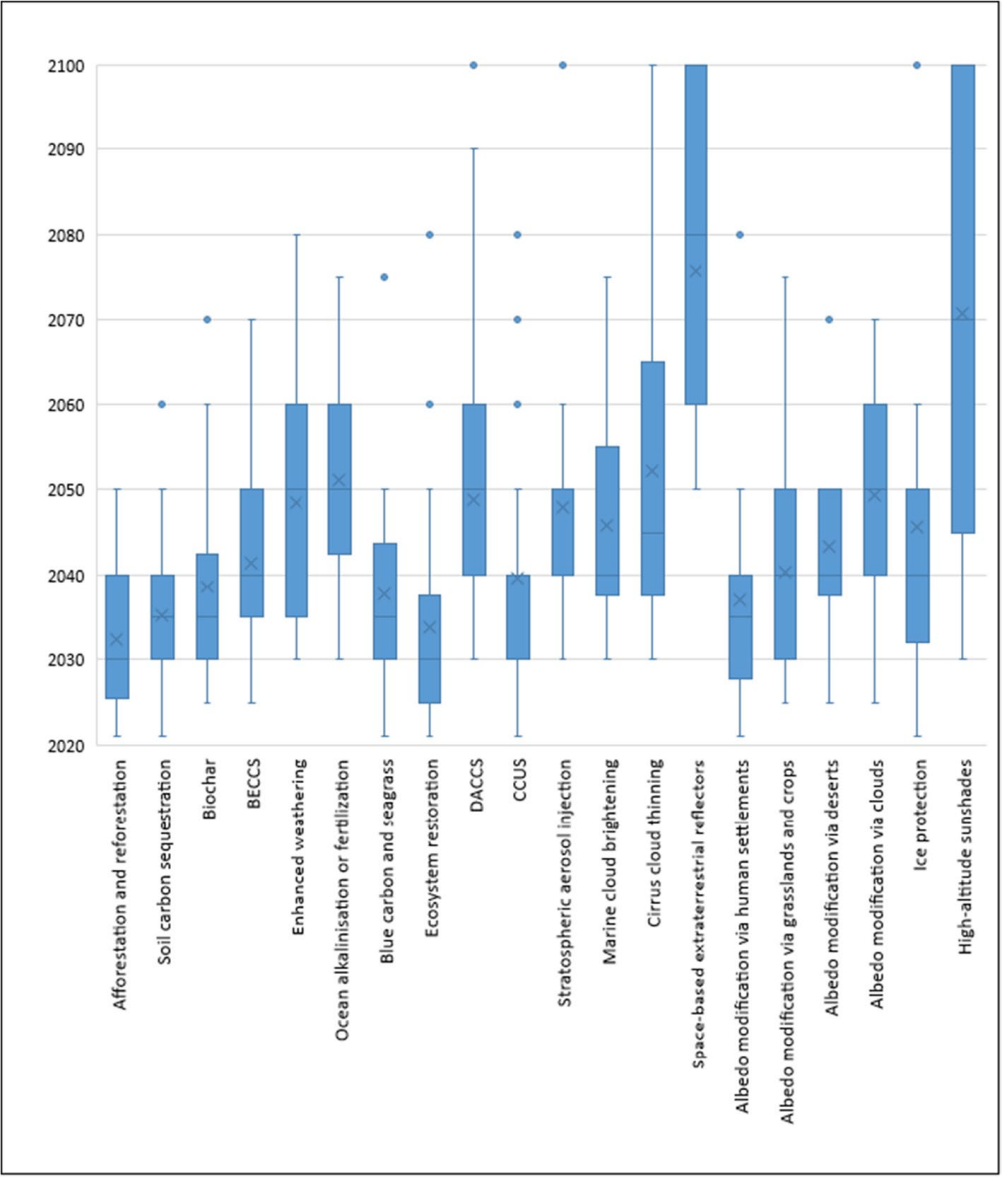
Expert perceptions about the scaling, commercialization, and deployment of negative-emissions and solar-geoengineering options. Source: Authors. The figure shows answers (*N* = 61 respondents) to the question “By what year (between now and 2100) would you expect each of the following options to achieve widespread deployment (e.g., a market share of 20%), enter 0 for never?” Experts were left to self-define how they interpreted “market share.” Bars within the boxes represent the median, while the mean is denoted by an “X.” Outliers are presented as dots. The full data tables behind this figure are presented in Appendix 3. BECCS, bioenergy with carbon capture and storage. DACCS, direct air capture with carbon storage. CCUS, carbon capture utilization and storage. Given the predominance of “never” being answered by our experts for several of these options, especially SRM ones, the estimates for certain technologies are only able to draw on responses from a smaller subset of data (see Appendix 3 for further detail). For technologies where “never” responses accounted for, a majority are detailed in the paragraph directly below, and the ranges above should best be interpreted as “optimistic” cases for deployment, that is, if deployment at scale actually comes to pass

The results, shown in Fig. 6, clearly depict three groupings of options that our experts believed would achieve near-term deployment (by 2035), mid-term deployment (by 2055), and long-term deployment (by 2056 or after). Using the median estimates (provided in full in Appendix 3), the only options that our experts suggested would achieve widespread near-term deployment within the next decade are afforestation and reforestation (2030) and ecosystem restoration (2030). A second, much larger class of options was deemed to reach deployment in the mid-term: soil carbon sequestration (2035), albedo modification via human settlements (2035), blue carbon and seagrass (2035), biochar (2035), carbon capture utilization and storage (2040), albedo modification via grasslands and crops (2040), bioenergy with carbon capture and storage (2040), albedo modification via deserts (2040), marine cloud brightening (2040), ice protection (2040), stratospheric aerosol injection (2040), cirrus cloud thinning (2045), enhanced weathering (2050), albedo modification via clouds (2050), direct air capture and storage (2050), and ocean alkalinization or fertilization (2050). A final class comprised of two options was envisioned to reach deployment only in the longer-term (if even then): high-altitude sunshades (2070) and space-based reflectors (2080). We also highlight that, if one focuses on the CDR options, which appear on the left side of Fig. 6, the much wider range of options like DACCS and enhanced weathering offers further evidence of the uncertainty around when, if ever, they might be deployed at scale.

Underlying the data in Fig. 6 were also suggestions from numerous experts that some of the options would *never* achieve widespread deployment. This even occurred for options such as afforestation and reforestation (7.5% of respondents) and soil carbon sequestration (12.2%), which were deemed feasible in the near-term by the expert consensus. Expert assessments for “never” achieving deployment were much higher for a fairly large number of options, each with 50% or more of respondents suggesting “never”:

- Albedo modification from human settlements (51.2%);
- Ice protection (54.8%);
- Stratospheric aerosol injection (65.3%);
- Marine cloud brightening (63.8%);
- Albedo modification by grasslands (68.3%);
- Albedo modification by deserts (78.0%);
- Cirrus cloud thinning (79.1%);
- Albedo modification by clouds (79.5%);
- Ocean alkalinization or fertilization (80.9%);
- High-altitude sunshades (82.9%);
- Space-based reflectors (84.4%).

This qualitative consensus among our experts may indeed suggest that such options be (at worst) ruled out of current climate discussions or (at best) treated with lower degrees of confidence and higher degrees of uncertainty.

### Concerns about composite risks and barriers

**Fig. 7:**
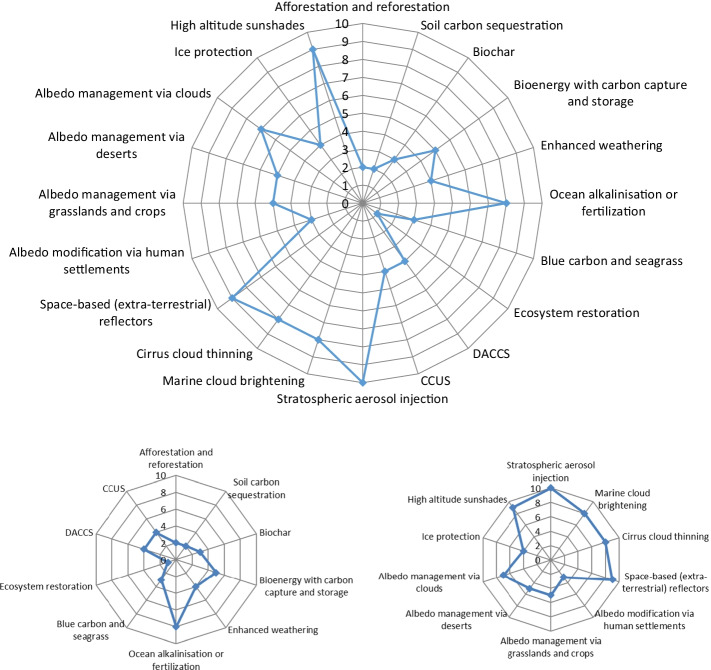
Expert perceptions about the composite risks facing negative-emissions and solar-geoengineering options. Source: Authors. The top panel depicts answers (*N* = 66 respondents) for all 20 options for the question “Each of the options below entails different social, economic, environmental, and even political risks. As of our evolving base of knowledge in 2021, how would you rate the risks of each of these options as they might be scaled up or engaged with in the future?” The higher the number, the riskier the option (medians shown, on a scale from 1 to 10). Experts were able to self-define what “composite risk” meant to them. The full data tables behind this figure are presented in Appendix 3. DACCS, direct air capture with carbon storage. CCUS, carbon capture utilization and storage. The bottom panel breaks the options apart in terms of CDR and SRM options, in order to underscore and illuminate the differences within these two categories

The final theme explored in our expert survey centered on concerns about the risks facing each of the climate pathways as well as the likelihood of different types of barriers. We tackled this theme in two ways. The first was by asking our experts to evaluate riskiness in a composite manner, that is, by thinking about how each option entails an amalgamation of risks spanning social, economic, environmental, and political dimensions. We asked our experts to rate how risky each option was (in these terms on a scale of one to ten) as of our knowledge base in 2021. Weighted-average responses again suggest a clustering of options (see Fig. 7). One set are perceived as low risk—that is scoring between a median of 0 and 4 within our survey exercise. This includes ecosystem restoration (the lowest composite risk score of 1.00), soil carbon sequestration (2.00), afforestation and reforestation (3.00), blue carbon and seagrass (3.00), biochar (3.00), albedo modification from human settlements (3.00), direct air capture (4.00), enhanced weathering (4.00), ice protection (4.00), and carbon capture and storage (4.00). One set are considered to be moderately risky with mean scores of 5 to 7: albedo modification via grasslands (5.00), albedo modification via deserts (5.00), bioenergy with carbon capture and storage (5.00), and albedo modification via clouds (7.00). And a final set are considered most risky with median composite scores of 8 and above: cirrus cloud thinning (8.00), marine cloud brightening (8.00), ocean alkalinization or fertilization (8.00), high-altitude sunshades (9.00), space-based reflectors (9.00), and stratospheric aerosol injection (10.00). In addition, looking at the bottom panel of Fig. 7, it becomes evident that our group of experts associated higher composite risks with almost all of the SRM options, with exception of ice protection and a few forms of albedo modification whereas more favorable views were held towards CDR options, with the notable exception of ocean alkalinization or fertilization.

The second way we explored this theme asked experts about the prevalence of particular barriers that they thought were facing CDR and SRM options, drawn from our familiarity with the literature on barriers and centered on eight core types: technology upscaling and readiness, (Buck 2019; National Research Council 2015; National Academies of Sciences Engineering, and Medicine 2019) storage disposal constraints (especially permanence) (Honegger and Reiner 2017; EASAC 2018; National Research Council 2015), social acceptance and public perceptions (Shrum et al. 2020; Cox et al. 2021; Wibeck et al. 2015; Bertram and Merk 2020; Buck 2018), legal and regulatory obstacles (Armeni 2015; Brent et al. 2015/2016; Craik 2015; Fleurke 2016; Garg 2014), challenges to system integration (GESAMP 2019; Buck 2019; Jeffery et al. 2020; Pietzcker et al. 2017), financing (Buck, et al. 2020; International Energy Agency Greenhouse Gas Division, Element Energy, and Imperial College London 2021; Honegger et al. 2021c), sustainable business models and market viability (Parson and Buck 2020; Rickels et al. 2020; Fuss et al. 2018), and risks to the environment or planetary health (Anderson and Peters 2016; Buck 2016; Obersteiner et al. 2018; Russell et al. 2012). A final category of “other factors” was meant to capture barriers that did not fit into these eight categories. As Table 3 indicates, all examined options had at least *some* barriers and many had multiple barriers—cirrus cloud thinning, high-altitude sunshades, and space-based reflectors were identified as having significant barriers in more than *half* of the barrier categories, as evidenced by the number of red and/or dark-yellow boxes. Conversely, options such as afforestation and reforestation, soil carbon sequestration, biochar, blue carbon and seagrass, ecosystem restoration, and albedo modification via human settlements were seen as having the fewest collective barriers, highlighted by the prevalence of green and pale-yellow boxes. Thus, if one looks for collections of red for a given option (e.g., for high-altitude sunshades and space-based reflectors), our findings signal the need to proceed with great caution. Conversely, options like ecosystem restoration, albedo modification via human settlements, soil carbon sequestration, and biochar are more of a “go” and involve less caution.

**Table 3:**
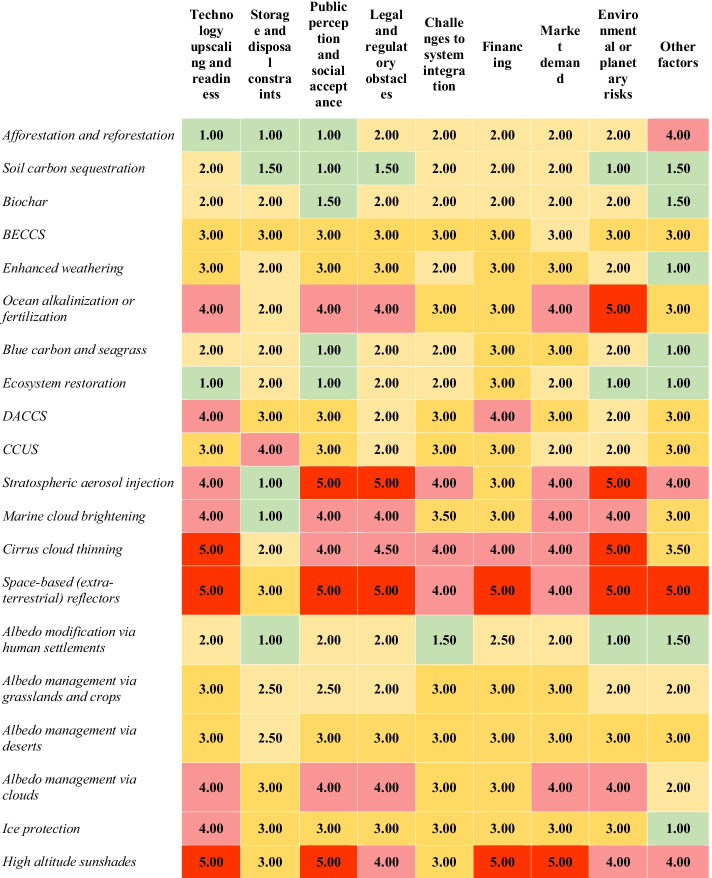
Expert perceptions about the salience of barriers facing negative-emissions and solar-geoengineering options

Source: Authors. Note: *BECCS*, bioenergy with carbon capture and storage. *DACCS*, direct air capture with carbon capture and storage. *CCUS*, carbon capture utilization and storage. The table depicts the results for the question (*N* = 69 respondents) “What do you see as the main potential barriers for the deployment of different CDR and SRM options at the global scale? Barriers were ranked as equally important. “Other factors” includes any barrier not explicitly listed. (Note: 1 = no/weak barrier, 5 = strong barrier). Median scores ranging from 1.00 to 1.99 are highlighted in pale green; those from 2.00 to 2.99 in pale yellow; from 3.00 to 3.99 in dark yellow; from 4.00 to 4.50 in light red, and 4.51 to 5.00 in dark red. The full data supporting this table is presented in Appendix 3

In terms of type, *technical*-related barriers such as upscaling, storage, and system integration (the far left two columns of Table 2 plus the column in the middle) were seen as significant for some options—notably ocean alkalinization or fertilization, direct air capture, marine cloud brightening, cirrus cloud thinning, space-based reflectors, albedo management via clouds and high-altitude sunshades. But *non-technical* barriers arose as significant for options such as ocean alkalinization or fertilization (environmental and planetary risk, social acceptance, legal and regulatory challenges), stratospheric aerosol injection (environmental and planetary risk, social acceptance, legal and regulatory barriers), marine cloud brightening (environmental and planetary risk, legal and regulatory barriers), cirrus cloud thinning (environmental and planetary risk, social acceptance, legal and regulatory barriers), space-based reflectors (environmental and planetary risk, social acceptance, legal and regulatory barriers, financing, market demand, other factors) and high-altitude sunshades (social acceptance, legal and regulatory barriers, financing, market viability). This finding validates research and policy focusing well beyond traditional concerns of technology deployment (e.g., beyond basic research and development) to broader themes of acceptance, governance, policy, and markets.

## Discussion and conclusion

Negative emissions and solar geoengineering options may contribute towards achieving climate and energy targets but, based on a large expert survey, their future feasibility remains highly contested. Unlike other elicitation processes where experts are more positive or have high expectations about novel options such as hydrogen fuel cells (Schmidt et al. 2017), solar energy (Lam et al. 2018; Verdolini et al. 2015), or nuclear power (Usher and Strachan 2013), our results are more critical and cautionary. Although a strong consensus of our experts considered negative emissions to be necessary to meet a 1.5C or 2C target of temperature change, this finding was reversed for solar geoengineering, with a supermajority of experts arguing that those options are not needed.

When put into distinct portfolios organized by a forced hierarchy of prioritization, our experts supported afforestation and reforestation, ecosystem restoration, and soil carbon sequestration as the most optimal negative emissions options and albedo modification via human settlements, albedo management via grasslands and crops, and marine cloud brightening as the most desirable solar geoengineering options (see Fig. 2 above). This contrasts with the least favored options including ocean alkalinization or fertilization, blue carbon and seagrass, and enhanced weathering along with space-based reflectors, high-altitude sunshades, and albedo management via clouds (see Fig. 2 above). This thinking aligns with the expected potential these options have by our experts, with afforestation and reforestation, soil carbon sequestration, and bioenergy with carbon capture and storage expected to have the most near-term emissions-reductions potential by 2050 (looking at the median values); stratospheric aerosol injection, albedo management via crops, and space-based reflectors are identified with a mean potential to reduce temperature the most, a finding that has not yet made it into Integrated Assessment Modeling, which has not by and large included solar geoengineering options in their technology portfolios.

This finding is salient not only for its substantive implications about the desirability of options, it also may reveal aspects of expert knowledge itself across the 20 options investigated. One potentially revealing finding is that experts agree most on the potential of afforestation and reforestation, and they have more favorable opinions about CDR than on solar geoengineering. This positive stance towards carbon removal could reflect that experts have a more hands-on experience with trees and forests than with many of the other more high-tech and remote options. Familiarity with options becomes a proxy for positive opinions about them. Furthermore, it is telling that no respondents gave estimations or opinions across all 20 options and most concentrated on only a handful of options. A deeper issue is that few experts had expert knowledge on all different technologies—one would not expect an enhanced weathering scientist to know much about marine cloud brightening, nor a stratospheric aerosol injection modeler to predict the cost of soil carbon storage in 2050.

The implication is that none of our experts feel like they have sufficient knowledge or insight across the 20 approaches elicited. This also makes sense intuitively. One would have to be rather versatile to keep abreast of such a range of so different technologies and approaches, i.e., to be knowledgeable about the mitigation potential/temperature reduction potential of 20 different techniques. In this vein, the results show that experts are careful to specify the limits of their expertise. We fully acknowledge that undertaking an analysis of how expert opinion varies by gender, experience, training, occupation, etc. would be very valuable for future research efforts and would also push future research towards state-of-the-art standards for both expert elicitation as well as multi-criteria decision-making (Keeney and Raiffa 1993; Verdolini et al. 2020).

Our expert data produces cogent findings about expected costs for negative emissions technologies, especially as we opt to focus on the median rather than mean values, in view of the variability and prominence of a couple outliers within the data. Within the negative-emissions category, afforestation and reforestation ($5–50, mean of $27.50), soil carbon sequestration ($0–50, average of $25), ecosystem restoration ($0–87.50, average of $43.75), and blue carbon and seagrass ($0–75, average of $37.50), and biochar ($20–100, average of $60) all have the lowest expected costs per ton removed by 2050. This contrasts noticeably with the expected costs for bioenergy with carbon capture and storage ($75–200, average of $137.50), enhanced weathering ($30–200, average of $115), ocean alkalinization or fertilization ($50–225, average of $137.50), direct air capture ($100–500, average of $300), and carbon capture utilization and storage ($50–200 to, average of $125). These latter options, especially direct air capture, could potentially be priced out of any competitive or affordable carbon market in 2050, even if the price of carbon were to surpass $100 per ton—depending on where their costs ultimately fall within this range. What is more, the degree to which the range between the minimum and maximum estimates varies across the options highlights the uncertainty—or construed more positively, the overall development potential—that attends to some of them, most notably, those of a more engineered nature such as direct air capture, carbon capture utilization and storage, and enhanced weathering.

Moreover, the comparative efficacy of the more affordable options becomes striking when compared to the others: using the mean numbers per ton of carbon removed (see Appendix 3 for all underlying data), soil carbon sequestration would be about 60 times more cost effective than bioenergy with carbon capture and storage. Our expert survey also finds that ecosystem restoration would be about 18 times more cost effective than carbon capture utilization and storage, and blue carbon and seagrass to be about 35 times more cost effective than direct air capture. Using the median numbers, soil carbon sequestration could be about 5.5 times more cost effective than bioenergy with carbon capture and storage; ecosystem restoration could be about 3 times more cost effective than carbon capture utilization and storage; and blue carbon and seagrass could be 8 times more cost effective than direct air capture.

It is not only economic cost that could rule some options out—the timing and likely commercialization of different innovations could also delay climate options, even some of the more cost-effective ones. Only the options of afforestation and reforestation (2030) and ecosystem restoration (2030) were expected to be widely deployed (e.g., surpassing a 20% market share) in the next decade. Much-discussed options such as carbon capture utilization and storage, bioenergy with carbon capture and storage, direct air capture, and virtually all solar-geoengineering options are not expected to achieve large-scale deployment until at least 2040, and some options were identified as not reaching deployment until 2050 at the earliest. This positions a preponderance of options as out of reach and unavailable for use within the next *two decades*. A majority of experts even suggested that some options—especially albedo modification by deserts, cirrus cloud thinning, albedo modification by clouds, ocean alkalization or fertilization, high-altitude sunshades, and space-based reflectors—would *never* reach commercialization.

Notably, options that have desirable attributes in terms of the portfolio ranking undertaken by our experts or affordable costs per ton still involve risks and barriers. Ecosystem restoration, soil sequestration, and afforestation and reforestation were seen as the least risky negative emissions; interestingly, some solar geoengineering options were seen as low risk, notably albedo modification via human settlements and via grasslands. The options with the highest perceived risks were ocean alkalization or fertilization, space-based reflectors, and stratospheric aerosol injection. Additionally, afforestation and reforestation, soil carbon sequestration, biochar, blue carbon and seagrass, ecosystem restoration, and albedo modification via human settlements were seen as having the fewest collective barriers; this contrasts with cirrus cloud thinning, high-altitude sunshades, and space-based reflectors which were perceived as having the most significant barriers. Our study also points the way towards clearly desired and supported pathways—centering on ecosystem restoration, soil sequestration, afforestation and reforestation, biochar—with a rare combination of affordable cost, near- to mid-term commercialization, comparatively fewer risks, and minimal to only moderate barriers.

Ultimately, our combined expert judgments about necessity, desirability, efficacy, expectations, and risks reveal tensions between these attributes of climate options (some of the options expected to achieve affordable and cost-effective reductions either face persistent barriers or entail moderate to high risks). For instance, some options have strong potential to abate carbon or stabilize the climate but low desirability (stratospheric aerosol injection, bioenergy with carbon capture and storage), or are strongly desirable but have limited abatement potential (e.g. marine cloud brightening, blue carbon and seagrass). Ocean alkalinization or fertilization is seen to have high potential for sequestration but also high unknowns. This creates an immense challenge for regulation, policy, and governance, given that optimal policy mixes will be forever changing based not only on cost and performance but issues over efficacy, scaling, risks, and barriers. Policymakers should therefore consider forms of adaptive risk management (Sovacool et al. 2022) and multi-criteria risk assessment (Stirling 2006, 2010) whenever they consider deployment, even if only deployment of one option. All options, even the ones seen as most desirable and effective by our experts, have risks and barriers, making it difficult to predict diffusion and assess costs and benefits.

## Appendix

### Appendix 1. Expert survey guide. GENIE Project. May 2021

1. What is your name?
2. Do you think that we will need greenhouse gas removal (GGR) and/or carbon dioxide removal (CDR) technologies in order to meet a 1.5C or 2C target, or whatever temperature goal you would define as limiting climate change to a non-dangerous level? (Yes/No)
3. Do you think that we will need solar radiation management (SRM) technologies in order to meet a 1.5C or 2C target, or whatever temperature goal you would define as limiting climate change to a non-dangerous level? (Yes/No)
4. The literature on energy and climate policy often discusses the following GGR and CDR options. Please rank them against each other in order of your preference. (You can rank them by inputting numbers, or by dropping and dragging each option into a ranked position).
  Afforestation and reforestation
  Soil carbon sequestration
  Biochar
  Bioenergy with carbon capture and storage (BECCS)
  Enhanced weathering
  Ocean alkalinization or fertilization
  Blue carbon and seagrass
  Ecosystem restoration
  Direct air capture and storage (DACCS)
  Carbon capture utilization and storage (CCUS)
5. The literature often discusses the following SRM options. Please rank them against each other in order of your preference. (You can rank them by inputting numbers, or by dropping and dragging each option into a ranked position).
  Stratospheric aerosol injection
  Marine cloud brightening
  Cirrus cloud thinning
  Space-based (extra-terrestrial) reflectors
  Albedo modification via human settlements
  Albedo management via grasslands and crops
  Albedo management via deserts
  Albedo management via clouds
  Ice protection
  High altitude sunshades
6. Another way of prioritizing options is to quantify them. By the year 2050, how many gigatons of carbon dioxide equivalent do you expect each of the following options to reduce, displace, or avoid? (You can input numbers with decimals to convey reductions in megatons; however, you do not have to provide numbers for all options, i.e., give answers for only those options you feel you have enough expertise to answer)
  Afforestation and reforestation
  Soil carbon sequestration
  Biochar
  Bioenergy with carbon capture and storage (BECCS)
  Enhanced weathering
  Ocean alkalinization or fertilization
  Blue carbon and seagrass
  Ecosystem restoration
  Direct air capture and storage (DACCS)
  Carbon capture utilization and storage (CCUS)
7. Similarly, another way of considering options is according to their costs of carbon removal. The uncertainties are obviously very large, so providing a range of estimations is fine. How would you estimate the range of costs per metric ton of carbon dioxide avoided by 2050? If you do not know, or do not have sufficient expertise to answer for a given option, you can leave it blank.

8. In terms of feasibility, by the year 2050, how much global warming or climate change (in degrees Celsius) do you expect each of these options to achieve reducing or addressing?
  Stratospheric aerosol injection
  Marine cloud brightening
  Cirrus cloud thinning
  Space-based (extra-terrestrial) reflectors
  Albedo modification via human settlements
  Albedo management via grasslands and crops
  Albedo management via deserts
  Albedo management via clouds
  Ice protection
  High-altitude sunshades
9. Each of the options below entails different social, economic, environmental, and even political risks. As of our evolving base of knowledge in 2021, how would you rate the risks of each of these options as they might be scaled up or engaged with in the future?

10. By what year (between now and 2100) would you expect each of the following options to achieve widespread deployment (e.g., a market share of 20%), enter 0 for never?
  Afforestation and reforestation
  Soil carbon sequestration
  Biochar
  Bioenergy with carbon capture and storage (BECCS)
  Enhanced weathering
  Ocean alkalinisation or fertilization
  Blue carbon and seagrass
  Ecosystem restoration
  Direct air capture and storage (DACCS)
  Carbon capture utilization and storage (CCUS)
  Stratospheric aerosol injection
  Marine cloud brightening
  Cirrus cloud thinning
  Space-based (extra-terrestrial) reflectors
  Albedo modification via human settlements
  Albedo management via grasslands and crops
  Albedo management via deserts
  Albedo management via clouds
  Ice protection
  High-altitude sunshades
11. Finally, what do you see as the main potential barriers for the deployment of different CDR and SRM options at the global scale? Barriers can be ranked as equally important. “Other factors” includes any barrier not explicitly listed. (Note: 1 = no/weak barrier, 5 = strong barrier).

### Appendix 2. List of experts that completed our elicitation exercise

**Table:**

Name	Actor type	Gender	Country	Institution
[Anonymous aerospace engineer]	Private sector + industrial associations	Male	Germany	[Aerospace and space systems company focusing on integrated spacecraft]
Asayama, Shinichiro	Government + intergovernmental organizations	Male	Japan	National Institute for Environmental Studies
Bazilian, Morgan	Universities + research institutes	Male	USA	Colorado School of Mines
Bellamy, Rob	Universities + research institutes	Male	UK	University of Manchester
Beuttler, Christoph	Private sector + industrial associations	Male	Switzerland	Climeworks
Boettcher, Miranda	Universities + research institutes	Female	Germany	Institute for Advanced Sustainability Studies (IASS)
Brauer, Uwe	Private sector + industrial associations	Male	Germany	Planetary Sunshade Foundation
Briggs, Chad	Universities + research institutes	Male	USA	University of Alaska, Anchorage
Buck, Holly Jean	Universities + research institutes	Female	USA	University at Buffalo
Burns, Wil	Universities + research institutes	Male	USA	American University
Centers, Ross	Private sector + industrial associations	Male	Germany	Planetary Sunshades
Chalecki, Beth	Universities + research institutes	Female	USA	University of Nebraska Omaha
Chavez, Anthony E	Universities + research institutes	Male	USA	Northern Kentucky University
Cobo Gutiérrez, Selene	Universities + research institutes	Female	Switzerland	ETH Zurich
Cox, Emily	Universities + research institutes	Female	UK	Cardiff University
Delina, Laurence	Universities + research institutes	Male	Hong Kong	Hong Kong University of Science and Technology
Dooley, Kate	Universities + research institutes	Female	Australia	University of Melbourne
Draper, Kathleen	Civil society	Female	USA	International Biochar Initiative
Elliott, David	Universities + research institutes	Male	UK	The Open University
Erbay, Yorukcan	Private sector + industrial associations	Male	UK	Element Energy
Felgenhauer, Tyler	Universities + research institutes	Male	USA	Duke University
Florin, Marie-Valentine	Civil society	Female	Switzerland	International Risk Governance Council (IRGC)
Forster, Piers	Universities + research institutes	Male	UK	University of Leeds
Fuhrman, Jay	Government + intergovernmental organizations	Male	USA	Pacific Northwest National Laboratory (PNNL)
Fuss, Sabine	Universities + research institutes	Female	Germany	Mercator Research Institute on Global Commons and Climate Change (MCC)
Gambhir, Ajay	Universities + research institutes	Male	UK	Imperial College London
Geden, Oliver	Government + intergovernmental organizations	Male	Germany	German Institute for International and Security Affairs (SWP)
Ghosh, Arunabha	Civil society	Male	India	Council on Energy, Environment and Water (CEEW)
Grant, Neil	Universities + research institutes	Male	UK	Imperial College London
Haigh, Joanna	Universities + research institutes	Female	UK	Imperial College London/Grantham Institute
Hamilton, Clive	Universities + research institutes	Male	Australia	Charles Stewart University
Hawkes, Adam D	Universities + research institutes	Male	UK	Imperial College London
Heap, Richard	Civil society	Male	UK	Carbon Removal Centre, Foresight Transitions
Herzog, Howard	Universities + research institutes	Male	USA	MIT
Heyen, Daniel	Universities + research institutes	Male	Germany	TU Kaiserslautern (formerly ETHZ)
Horton, Joshua B	Universities + research institutes	Male	USA	Harvard University
Irvine, Pete	Universities + research institutes	Male	UK	UCL
Jinnah, Sikina	Universities + research institutes	Female	USA	UC Santa Cruz
Kammen, Daniel	Universities + research institutes	Male	USA	UC Berkeley
Keller, David	Universities + research institutes	Male	Germany	GEOMAR—Helmholtz Centre for Ocean Research Kiel
Keller, Klaus	Universities + research institutes	Male	USA	Penn State University
Kravitz, Ben	Universities + research institutes	Male	USA	Indiana University
Kuswanto, Heri	Universities + research institutes	Male	Indonesia	Institut Teknologi Sepuluh Nopember
Lehmann, Johannes	Universities + research institutes	Male	USA	Cornell University
Lin, Albert	Universities + research institutes	Male	USA	UC Davis
MacMartin, Doug	Universities + research institutes	Male	USA	Cornell University
Mahajan, Aseem	Universities + research institutes	Male	USA	Harvard University
Malik, Abdul	Universities + research institutes	Male	Saudi Arabia	King Abdullah University of Science and Technology (formerly Grantham Institute)
Mengis, Nadine	Universities + research institutes	Female	Germany	GEOMAR—Helmholtz Centre for Ocean Research Kiel
Michaelowa, Axel	Universities + research institutes/private sector + industrial associations	Male	Switzerland	University of Zurich/Perspectives Climate Group
Moreno-Cruz, Juan	Universities + research institutes	Male	Canada	University of Waterloo
Morrow, David	Universities + research institutes	Male	USA	American University
Odoulami, Romaric	Universities + research institutes	Male	South Africa	University of Cape Town
Pidgeon, Nick	Universities + research institutes	Male	UK	Cardiff University
Pongratz, Julia	Universities + research institutes	Female	Germany	University of Munich
Preston Aragonès, Mark	Civil society	Male	Norway	Bellona Foundation
Raimi, Kaitlin T	Universities + research institutes	Female	USA	University Michigan
Reynolds, Jesse	Universities + research institutes	Male	USA/Netherlands	UCLA/Independent Consultant
Rickels, Wilfried	Universities + research institutes	Male	Germany	Kiel Institute
Robock, Alan	Universities + research institutes	Male	USA	Rutgers University
Schleussner, Carl	Civil society	Male	USA	Climate Analytics
Simonelli, Lucia	Civil society	Female	USA	Carbon 180
Smith, Pete	Universities + research institutes	Male	UK	University of Aberdeen
Smith, Wake	Universities + research institutes	Male	USA	Harvard University
Spangenberg, Joachim	Universities + research institutes	Male	Germany	Sustainable Europe Research Institute SERI Germany
Stephens, Jennie	Universities + research institutes	Female	USA	Northeastern University
Stoefs, Wijnand	Civil society	Male	Belgium	Carbon Market Watch
Sugiyama, Masahiro	Universities + research institutes	Male	Japan	University of Tokyo
Sunny, Nixon	Universities + research institutes	Male	UK	Imperial College London
van Vuuren, Detlef	Government + intergovernmental organizations	Male	Netherlands	PBL Netherlands Environmental Assessment Agency
Victor, David	Universities + research institutes	Male	USA	UC San Diego
Wolske, Kimberly S	Universities + research institutes	Female	USA	University Chicago
Wood, Robert	Universities + research institutes	Male	USA	University of Washington
Workman, Mark	Universities + research institutes	Male	UK	Energy Futures Lab, Imperial College London

### Appendix 3 Data tables

Q4: Ranking of CDR options

**Table:**

Statistics											
		Afforestation and reforestation	Soil carbon sequestration	Biochar	BECCS	Enhanced weathering	Ocean alkalinization or fertilization	Blue carbon and seagrass	Ecosystem restoration	DACCS	CCUS
*N*	Valid	70	66	68	68	69	65	67	68	69	69
	Missing	4	8	6	6	5	9	7	6	5	5
Mean		2.61	3.76	5.74	5.43	6.20	8.97	6.43	3.82	5.39	6.04
Median		2.00	3.00	6.00	5.50	6.00	10.00	6.00	3.00	6.00	7.00
Std. deviation		1.627	2.000	2.027	2.301	2.272	1.457	2.169	2.648	2.824	3.440
Minimum		1	1	1	1	1	4	1	1	1	1
Maximum		8	10	10	10	10	10	10	10	10	10

Q5: Ranking of SRM options

**Table:**

Statistics											
		Stratospheric aerosol injection	Marine cloud brightening	Cirrus cloud thinning	Space-based (extra-terrestrial) reflectors	Albedo modification via human settlements	Albedo management via grasslands and crops	Albedo management via deserts	Albedo management via clouds	Ice protection	High altitude sunshades
*N*	Valid	60	60	58	59	60	58	58	57	59	57
	Missing	14	14	16	15	14	16	16	17	15	17
Mean		5.02	4.52	5.52	7.54	3.40	4.05	5.76	6.12	4.58	7.54
Median		5.00	4.50	6.00	9.00	3.00	3.50	5.50	6.00	4.00	8.00
Std. deviation		3.798	2.296	2.319	2.867	2.395	2.409	2.401	1.722	2.972	2.188
Minimum		1	1	2	1	1	1	1	2	1	1
Maximum		10	10	10	10	10	10	10	10	10	10

Q6: Gigatons of CO2 reduction

**Table:**

Statistics											
		Afforestation and reforestation	Soil carbon sequestration	Biochar	BECCS	Enhanced weathering	Ocean alkalinization or fertilization	Blue carbon and seagrass	Ecosystem restoration	DACCS	CCUS
*N*	Valid	36	32	30	35	26	20	20	25	36	27
	Missing	38	42	44	39	48	54	54	49	38	47
Mean		61.250	36.05159	40.3367	48.783	13.377	58.910	26.3400	35.9884	38.1489	24.55041
Median		2.000	2.00000	0.7500	2.000	0.750	0.000	0.3500	1.0000	1.0000	1.00000
Std. deviation		192.8474	103.286856	127.75054	176.2202	34.0384	259.1799	111.57466	123.72854	167.06060	96.925311
Minimum		0.5	0.000	0.00	0.0	0.0	0.0	0.00	0.01	0.00	0.000
Maximum		1000.0	500.000	500.00	1000.0	116.0	1160.0	500.00	580.00	1000.00	500.000

Q7: Minimum and maximum cost per tCO2

**Table:**

Statistics											
		Afforestation and reforestation—minimum	Afforestation and reforestation—maximum	Soil carbon sequestration—minimum	Soil carbon sequestration—maximum	Biochar—minimum	Biochar—maximum	BECCS—minimum	BECCS—maximum	Enhanced weathering—minimum	Enhanced weathering—maximum
*N*	Valid	36	33	31	28	28	26	31	32	21	20
	Missing	38	41	43	46	46	48	43	42	53	54
Mean		9.17	111.67	6.61	56.79	54.50	3978.12	102.77	3665.34	87.43	5180.30
Median		5.00	50.00	0.00	50.00	20.00	100.00	75.00	200.00	30.00	200.00
Std. deviation		16.797	178.482	14.854	42.496	186.131	19,585.767	173.098	17,664.862	212.074	22,318.898
Minimum		0	0	0	0	0	20	5	15	0	30
Maximum		100	1000	75	150	1001	100,001	1001	100,001	1001	100,001

**Table:**

Statistics											
		Ocean alkalinization or fertilization—minimum	Ocean alkalinization or fertilization—maximum	Blue carbon and seagrass—minimum	Blue carbon and seagrass—maximum	Ecosystem restoration—minimum	Ecosystem restoration—maximum	DACCS—minimum	DACCS—maximum	CCUS—minimum	CCUS—Maximum
*N*	Valid	13	12	13	11	18	18	39	37	19	18
	Missing	61	62	61	63	56	56	35	37	55	56
Mean		123.92	8579.25	35.00	161.82	9.17	636.67	191.31	6633.81	152.95	11,326.50
Median		50.00	225.00	0.00	75.00	0.00	87.50	100.00	500.00	50.00	200.00
Std. deviation		266.515	28,790.876	65.574	286.525	16.292	2337.815	228.278	22,780.219	301.967	32,260.742
Minimum		5	50	0	5	0	0	30	100	0	75
Maximum		1001	100,001	200	1000	50	10,000	1001	100,001	1001	100,001

Q8: Feasible temperature reduction

**Table:**

Statistics											
		Stratospheric aerosol injection	Marine cloud brightening	Cirrus cloud thinning	Space-based (extra-terrestrial) reflectors	Albedo modification via human settlements	Albedo management via grasslands and crops	Albedo management via deserts	Albedo management via clouds	Ice protection	High-altitude sunshades
*N*	Valid	38	32	29	32	29	29	28	29	30	30
	Missing	36	42	45	42	45	45	46	45	44	44
Mean		0.97	0.44	0.34	0.66	0.45	0.90	0.39	0.34	0.37	0.33
Median		0.00	0.00	0.00	0.00	0.00	0.00	0.00	0.00	0.00	0.00
Std. deviation		1.700	1.435	1.495	2.223	1.502	3.098	1.524	1.495	1.474	1.470
Minimum		0	0	0	0	0	0	0	0	0	0
Maximum		8	8	8	10	8	15	8	8	8	8

Q9: Riskiness

**Table:**

Statistics											
		Afforestation and reforestation	Soil carbon sequestration	Biochar	BECCS	Enhanced weathering	Ocean alkalinization or fertilization	Blue carbon and seagrass	Ecosystem restoration	DACCS	CCUS
*N*	Valid	63	57	56	58	58	51	51	56	56	56
	Missing	11	17	18	16	16	23	23	18	18	18
Mean		2.92	2.58	3.29	5.66	4.43	7.98	3.25	2.09	4.36	4.82
Median		3.00	2.00	3.00	5.00	4.00	8.00	3.00	1.00	4.00	4.00
Std. deviation		1.649	1.625	1.755	2.140	1.874	1.913	1.998	1.468	2.119	2.684
Minimum		1	1	1	1	1	2	1	1	1	1
Maximum		8	8	9	10	9	10	9	6	9	10

**Table:**

Statistics											
		Stratospheric aerosol injection	Marine cloud brightening	Cirrus cloud thinning	Space-based (extra-terrestrial) reflectors	Albedo modification via human settlements	Albedo management via grasslands and crops	Albedo management via deserts	Albedo management via clouds	Ice protection	High-altitude sunshades
*N*	Valid	56	48	46	46	48	44	42	41	46	41
	Missing	18	26	28	28	26	30	32	33	28	33
Mean		8.86	7.83	7.74	8.00	3.79	4.68	5.43	6.76	5.17	7.59
Median		10.00	8.00	8.00	9.00	3.00	5.00	5.00	7.00	4.00	9.00
Std. deviation		1.911	1.655	1.731	2.494	2.705	2.639	2.577	2.278	2.807	2.480
Minimum		1	4	4	1	1	1	2	2	1	1
Maximum		10	10	10	10	10	10	10	10	10	10

Q10: Market and deployment

**Table:**

Statistics											
		Afforestation and reforestation	Soil carbon sequestration	Biochar	BECCS	Enhanced weathering	Ocean alkalinization or fertilization	Blue carbon and seagrass	Ecosystem restoration	DACCS	CCUS
*N*	Valid	49	43	34	42	28	9	24	37	44	38
	Missing	25	31	40	32	46	65	50	37	30	36
Mean		2032.4286	2035.1628	2038.6471	2041.3333	2048.4286	2051.1111	2037.7500	2033.8108	2048.8864	2039.6316
Median		2030.0000	2035.0000	2035.0000	2040.0000	2050.0000	2050.0000	2035.0000	2030.0000	2050.0000	2040.0000
Std. deviation		8.77971	8.28921	10.02688	10.91132	13.69896	13.86943	11.54293	11.70194	14.63226	12.28143
Minimum		2021.00	2021.00	2025.00	2025.00	2030.00	2030.00	2021.00	2021.00	2030.00	2021.00
Maximum		2050.00	2060.00	2070.00	2070.00	2080.00	2075.00	2075.00	2080.00	2100.00	2080.00

**Table:**

Statistics											
		Stratospheric aerosol injection	Marine cloud brightening	Cirrus cloud thinning	Space-based extraterrestrial reflectors	Albedo modification via human settlements	Albedo modification via grasslands and crops	Albedo modification via deserts	Albedo modification via clouds	Ice protection	High-altitude sunshades
*N*	Valid	17	17	9	7	20	13	9	8	19	7
	Missing	57	57	65	67	54	61	65	66	55	67
Mean		2047.9412	2045.8824	2052.2222	2075.7143	2036.9500	2040.3846	2043.3333	2049.3750	2045.6842	2070.7143
Median		2040.0000	2040.0000	2045.0000	2080.0000	2035.0000	2040.0000	2040.0000	2050.0000	2040.0000	2070.0000
Std. deviation		17.85872	12.02020	21.81042	19.88060	13.58201	13.61089	12.50000	14.25219	21.90396	29.78095
Minimum		2030.00	2030.00	2030.00	2050.00	2021.00	2025.00	2025.00	2025.00	2021.00	2030.00
Maximum		2100.00	2075.00	2100.00	2100.00	2080.00	2075.00	2070.00	2070.00	2100.00	2100.00

Q11—Barriers

Technology upscaling and readiness

**Table:**

Statistics											
		Afforestation and reforestation	Soil carbon sequestration	Biochar	BECCS	Enhanced weathering	Ocean alkalinization or fertilization	Blue carbon and seagrass	Ecosystem restoration	DACCS	CCUS
*N*	Valid	55	49	49	56	48	43	42	43	55	50
	Missing	19	25	25	18	26	31	32	31	19	24
Mean		1.47	2.10	2.51	3.18	3.31	4.05	2.45	1.86	4.05	3.30
Median		1.00	2.00	2.00	3.00	3.00	4.00	2.00	1.00	4.00	3.00
Std. deviation		0.940	1.262	1.157	0.974	1.274	0.950	1.194	1.207	1.026	1.035

**Table:**

Statistics											
		Stratospheric aerosol injection	Marine cloud brightening	Cirrus cloud thinning	Space-based extraterrestrial reflectors	Albedo modification via human settlements	Albedo modification via grasslands and crops	Albedo modification via deserts	Albedo modification via clouds	Ice protection	High-altitude sunshades
*N*	Valid	44	37	33	39	32	28	27	24	32	29
	Missing	30	37	41	35	42	46	47	50	42	45
Mean		3.84	4.11	4.55	4.82	2.16	2.86	3.15	4.21	3.56	4.48
Median		4.00	4.00	5.00	5.00	2.00	3.00	3.00	4.00	4.00	5.00
Std. deviation		1.328	0.966	0.794	0.451	1.194	1.239	1.167	0.884	1.268	0.911

Storage/disposal constraints

**Table:**

Statistics											
		Afforestation and reforestation	Soil carbon sequestration	Biochar	BECCS	Enhanced weathering	Ocean alkalinization or fertilization	Blue carbon and seagrass	Ecosystem restoration	DACCS	CCUS
*N*	Valid	49	44	44	55	40	36	35	40	54	49
	Missing	25	30	30	19	34	38	39	34	20	25
Mean		2.08	2.05	2.25	3.11	2.10	2.42	2.31	2.15	2.89	3.37
Median		1.00	1.50	2.00	3.00	2.00	2.00	2.00	2.00	3.00	4.00
Std. deviation		1.288	1.219	1.184	1.329	0.982	1.442	1.491	1.388	1.313	1.349

**Table:**

Statistics											
		Stratospheric aerosol injection	Marine cloud brightening	Cirrus cloud thinning	Space-based extraterrestrial reflectors	Albedo modification via human settlements	Albedo modification via grasslands and crops	Albedo modification via deserts	Albedo modification via clouds	Ice protection	High-altitude sunshades
*N*	Valid	28	22	19	22	19	18	18	17	19	19
	Missing	46	52	55	52	55	56	56	57	55	55
Mean		2.50	2.23	2.32	2.77	2.26	2.39	2.28	2.53	2.74	2.89
Median		1.00	1.00	2.00	3.00	1.00	2.50	2.50	3.00	3.00	3.00
Std. deviation		1.816	1.541	1.529	1.716	1.558	1.378	1.227	1.586	1.522	1.487

Public perception and social acceptance

**Table:**

Statistics											
		Afforestation and reforestation	Soil carbon sequestration	Biochar	BECCS	Enhanced weathering	Ocean alkalinization or fertilization	Blue carbon and seagrass	Ecosystem restoration	DACCS	CCUS
*N*	Valid	51	43	40	51	43	43	36	38	50	43
	Missing	23	31	34	23	31	31	38	36	24	31
Mean		1.75	1.58	1.90	3.39	2.67	4.37	1.64	1.21	2.72	2.86
Median		1.00	1.00	1.50	3.00	3.00	4.00	1.00	1.00	3.00	3.00
Std. deviation		1.074	0.879	1.150	1.185	1.063	0.691	0.961	0.474	0.948	1.082

**Table:**

Statistics											
		Stratospheric aerosol injection	Marine cloud brightening	Cirrus cloud thinning	Space-based extraterrestrial reflectors	Albedo modification via human settlements	Albedo modification via grasslands and crops	Albedo modification via deserts	Albedo modification via clouds	Ice protection	High-altitude sunshades
*N*	Valid	49	40	32	37	29	26	24	21	26	26
	Missing	25	34	42	37	45	48	50	53	48	48
Mean		4.82	3.95	4.16	4.59	2.07	2.50	3.17	3.76	2.54	4.42
Median		5.00	4.00	4.00	5.00	2.00	2.50	3.00	4.00	3.00	5.00
Std. deviation		0.441	0.846	0.920	0.599	1.193	1.273	1.239	1.136	1.174	0.758

Legal and regulatory obstacles

**Table:**

Statistics											
		Afforestation and reforestation	Soil carbon sequestration	Biochar	BECCS	Enhanced weathering	Ocean alkalinization or fertilization	Blue carbon and seagrass	Ecosystem restoration	DACCS	CCUS
*N*	Valid	52	42	42	49	42	40	35	37	49	43
	Missing	22	32	32	25	32	34	39	37	25	31
Mean		2.04	1.74	1.86	2.71	2.71	4.13	2.26	1.81	2.55	2.49
Median		2.00	1.50	2.00	3.00	3.00	4.00	2.00	2.00	2.00	2.00
Std. deviation		1.066	0.857	0.926	1.118	1.088	0.853	1.039	0.908	1.119	1.162

**Table:**

Statistics											
		Stratospheric aerosol injection	Marine cloud brightening	Cirrus cloud thinning	Space-based extraterrestrial reflectors	Albedo modification via human settlements	Albedo modification via grasslands and crops	Albedo modification via deserts	Albedo modification via clouds	Ice protection	High-altitude sunshades
*N*	Valid	45	38	32	37	27	27	26	22	28	27
	Missing	29	36	42	37	47	47	48	52	46	47
Mean		4.62	4.08	4.28	4.43	2.00	2.37	3.12	3.68	3.25	4.33
Median		5.00	4.00	4.50	5.00	2.00	2.00	3.00	4.00	3.00	4.00
Std. deviation		0.684	0.997	0.851	1.042	0.961	1.079	1.177	1.086	1.175	0.784

Challenges related to integration

**Table:**

Statistics											
		Afforestation and reforestation	Soil carbon sequestration	Biochar	BECCS	Enhanced weathering	Ocean alkalinization or fertilization	Blue carbon and seagrass	Ecosystem restoration	DACCS	CCUS
N	Valid	44	40	39	44	35	34	33	35	45	39
	Missing	30	34	35	30	39	40	41	39	29	35
Mean		2.09	2.40	2.08	2.77	2.43	2.97	2.00	2.09	2.87	2.72
Median		2.00	2.00	2.00	3.00	2.00	3.00	2.00	2.00	3.00	3.00
Std. deviation		1.178	1.317	0.984	1.198	1.195	1.547	1.146	1.222	1.290	1.213

**Table:**

Statistics											
		Stratospheric aerosol injection	Marine cloud brightening	Cirrus cloud thinning	Space-based extraterrestrial reflectors	Albedo modification via human settlements	Albedo modification via grasslands and crops	Albedo modification via deserts	Albedo modification via clouds	Ice protection	High-altitude sunshades
*N*	Valid	34	26	23	24	22	20	20	16	20	19
	Missing	40	48	51	50	52	54	54	58	54	55
Mean		3.76	3.38	3.61	3.50	1.95	2.80	2.80	3.00	2.90	3.26
Median		4.00	3.50	4.00	4.00	1.50	3.00	3.00	3.00	3.00	3.00
Std. deviation		1.478	1.359	1.438	1.642	1.253	1.322	1.240	1.414	1.334	1.284

Financing

**Table:**

Statistics											
		Afforestation and reforestation	Soil carbon sequestration	Biochar	BECCS	Enhanced weathering	Ocean alkalinization or fertilization	Blue carbon and seagrass	Ecosystem restoration	DACCS	CCUS
*N*	Valid	51	44	43	52	44	42	35	41	53	46
	Missing	23	30	31	22	30	32	39	33	21	28
Mean		2.33	2.50	2.53	2.98	3.27	3.48	2.89	2.61	3.89	3.22
Median		2.00	2.00	2.00	3.00	3.00	3.00	3.00	3.00	4.00	3.00
Std. deviation		1.071	1.110	1.099	1.260	1.107	1.153	1.132	1.222	1.138	1.209

**Table:**

Statistics											
		Stratospheric aerosol injection	Marine cloud brightening	Cirrus cloud thinning	Space-based extraterrestrial reflectors	Albedo modification via human settlements	Albedo modification via grasslands and crops	Albedo modification via deserts	Albedo modification via clouds	Ice protection	High-altitude sunshades
*N*	Valid	41	31	25	33	26	23	22	19	26	25
	Missing	33	43	49	41	48	51	52	55	48	49
Mean		2.73	3.29	3.72	4.52	2.54	2.87	3.41	3.63	3.23	4.12
Median		3.00	3.00	4.00	5.00	2.50	3.00	3.00	3.00	3.00	5.00
Std. deviation		1.397	1.296	1.242	1.121	1.363	1.254	1.221	1.065	1.275	1.130

Market demand

**Table:**

Statistics											
		Afforestation and reforestation	Soil carbon sequestration	Biochar	BECCS	Enhanced weathering	Ocean alkalinization or fertilization	Blue carbon and seagrass	Ecosystem restoration	DACCS	CCUS
*N*	Valid	49	42	42	48	39	35	35	39	47	41
	Missing	25	32	32	26	35	39	39	35	27	33
Mean		2.20	2.43	2.57	2.65	3.13	3.49	2.80	2.46	3.02	2.63
Median		2.00	2.00	2.00	3.00	3.00	4.00	3.00	2.00	3.00	2.00
Std. deviation		1.136	1.063	1.016	1.158	1.151	1.269	1.079	1.120	1.375	1.199

**Table:**

Statistics											
		Stratospheric aerosol injection	Marine cloud brightening	Cirrus cloud thinning	Space-based extraterrestrial reflectors	Albedo modification via human settlements	Albedo modification via grasslands and crops	Albedo modification via deserts	Albedo modification via clouds	Ice protection	High-altitude sunshades
*N*	Valid	33	26	24	28	25	22	21	18	22	21
	Missing	41	48	50	46	49	52	53	56	52	53
Mean		3.27	3.35	3.63	3.93	2.52	3.09	3.24	3.56	3.18	4.14
Median		4.00	4.00	4.00	4.00	2.00	3.00	3.00	4.00	3.00	5.00
Std. deviation		1.526	1.384	1.377	1.331	1.194	1.065	1.338	1.149	1.368	1.195

Environmental/planetary risks

**Table:**

Statistics											
		Afforestation and reforestation	Soil carbon sequestration	Biochar	BECCS	Enhanced weathering	Ocean alkalinization or fertilization	Blue carbon and seagrass	Ecosystem restoration	DACCS	CCUS
*N*	Valid	52	44	44	51	43	43	37	41	51	44
	Missing	22	30	30	23	31	31	37	33	23	30
Mean		2.08	1.34	2.07	3.31	2.49	4.30	1.92	1.22	2.02	2.43
Median		2.00	1.00	2.00	3.00	2.00	5.00	2.00	1.00	2.00	2.00
Std. deviation		1.152	0.568	1.208	1.273	1.121	0.939	1.064	0.571	0.990	1.283

**Table:**

Statistics											
		Stratospheric aerosol injection	Marine cloud brightening	Cirrus cloud thinning	Space-based extraterrestrial reflectors	Albedo modification via human settlements	Albedo modification via grasslands and crops	Albedo modification via deserts	Albedo modification via clouds	Ice protection	High-altitude sunshades
*N*	Valid	51	40	34	38	27	30	29	24	27	27
	Missing	23	34	40	36	47	44	45	50	47	47
Mean		4.41	4.13	4.29	4.18	1.52	2.50	3.34	3.83	2.96	3.93
Median		5.00	4.00	5.00	5.00	1.00	2.00	3.00	4.00	3.00	4.00
Std. deviation		0.963	0.853	0.871	1.249	0.753	1.358	1.446	1.129	1.126	1.238

Other factors

**Table:**

Statistics											
		Afforestation and reforestation	Soil carbon sequestration	Biochar	BECCS	Enhanced weathering	Ocean alkalinization or fertilization	Blue carbon and seagrass	Ecosystem restoration	DACCS	CCUS
*N*	Valid	20	14	12	15	13	11	8	12	17	15
	Missing	54	60	62	59	61	63	66	62	57	59
Mean		3.30	2.14	1.75	3.00	2.15	2.64	1.63	1.67	2.82	2.80
Median		4.00	1.50. an	1.50	3.00	1.00	3.00	1.00	1.00	3.00	3.00
Std. deviation		1.689	1.460	0.965	1.558	1.573	1.629	1.408	1.371	1.629	1.656

**Table:**

Statistics											
		Stratospheric aerosol injection	Marine cloud brightening	Cirrus cloud thinning	Space-based extraterrestrial reflectors	Albedo modification via human settlements	Albedo modification via grasslands and crops	Albedo modification via deserts	Albedo modification via clouds	Ice protection	High-altitude sunshades
*N*	Valid	15	7	6	7	4	4	4	3	5	4
	Missing	59	67	68	67	70	70	70	71	69	70
Mean		3.60	3.29	3.33	4.43	1.50	2.00	2.75	2.67	2.20	3.50
Median		4.00	3.00	3.50	5.00	1.50	2.00	3.00	2.00	1.00	4.00
Std. deviation		1.724	1.799	1.633	1.512	0.577	0.816	1.500	2.082	1.643	1.732

## Footnotes

1 Because the project was limited at 20 options, we had to combine some techniques that are sometimes treated as separate, e.g. ocean fertilization or alkalization, and afforestation with reforestation.
